# Microbe Finder (MiFi^®^): Implementation of an Interactive Pathogen Detection Tool in Metagenomic Sequence Data

**DOI:** 10.3390/plants10020250

**Published:** 2021-01-28

**Authors:** Andres S. Espindola, Kitty F. Cardwell

**Affiliations:** 1 Institute of Biosecurity and Microbial Forensics (IBMF), Oklahoma State University, Stillwater, OK 74078, USA; kitty.cardwell@okstate.edu; 2 Department of Entomology and Plant Pathology, Oklahoma State University, Stillwater, OK 74078, USA

**Keywords:** Microbe Finder (MiFi), diagnostics, nucleic acid, metagenomics, pathogens, taxonomy, sequencing, assay validation, sensitivity and specificity

## Abstract

Agricultural high throughput diagnostics need to be fast, accurate and have multiplexing capacity. Metagenomic sequencing is being widely evaluated for plant and animal diagnostics. Bioinformatic analysis of metagenomic sequence data has been a bottleneck for diagnostic analysis due to the size of the data files. Most available tools for analyzing high-throughput sequencing (HTS) data require that the user have computer coding skills and access to high-performance computing. To overcome constraints to most sequencing-based diagnostic pipelines today, we have developed Microbe Finder (MiFi^®^). MiFi^®^ is a web application for quick detection and identification of known pathogen species/strains in raw, unassembled HTS metagenomic data. HTS-based diagnostic tools developed through MiFi^®^ must pass rigorous validation, which is outlined in this manuscript. MiFi^®^ allows researchers to collaborate in the development and validation of HTS-based diagnostic assays using MiProbe™, a platform used for developing pathogen-specific e-probes. Validated e-probes are made available to diagnosticians through MiDetect™. Here we describe the e-probe development, curation and validation process of MiFi^®^ using grapevine pathogens as a model system. MiFi^®^ can be used with any pathosystem and HTS platform after e-probes have been validated.

## 1. Introduction

Rapid and accurate pathogen detection in plants and animals is critical to food security as well as public health. It is estimated that exotic animal and plant diseases cost United States agriculture billions each year [1]. With time and more trade, it is expected that the threat of invasive and exotic pathogens increases, requiring high throughput testing of agricultural imports. The lack of high throughput pathogen detection techniques at ports of entry and borders make some countries more vulnerable than others [2]. Not only do imported agricultural goods but also local trade have the potential to disseminate pathogens. Proactive measures to avoid the spread of diseases include extensive surveillance and testing, but these are limited by the cost and throughput capacity of current diagnostic technology. An ability to simultaneously screen for all possible pathogens per sample will enable more timely response, mitigation and management of potential plant, animal and human disease introductions and outbreaks.

Sequence-based detection technology has been explored recently by multiple plant quarantine agencies around the world [3,4]. Until recently, nucleic acid sequencing for diagnostics has been constrained by cost, data volume, and limited bioinformatic tools for analysis, creating a bottleneck to adoption by most diagnostic clinics. High-throughput sequencing (HTS) coupled with dedicated bioinformatic analysis promise to overcome the technical difficulties in detection. HTS-based diagnostics that target all pathogens in a given host can be used to declare a sample free-from-pathogen. MiFi^®^ is a bioinformatic graphical user interface built to close the gap between HTS and pathogen detection. MiFi^®^ uses, as a founding concept, the E-probe Diagnostics for Nucleic acid Analysis (EDNA) process [5,6] that queries the metagenome raw data of the sample tissue and all resident microbes, to identify known pathogen sequences. The MiFi^®^ platform comprises two parts. (1) MiProbe™, freely available to all, is a platform that houses tools for building, curating and validating microbe-specific e-probes [7]. MiFi^®^ e-probes are unique nucleic acid signature sequences carefully curated from across the pathogen genome. E-probes are used in silico to detect the presence or absence of one or more pathogens in metagenomic sequence data. MiProbe™ is a unique piece of bioinformatic software for diagnostics that, to the best of our knowledge, no other research team has developed. Other bioinformatic tools available such as Metaphlan, MEGAN, Kraken and QIIME have curated their own databases and made them available for researchers to use [8,9,10,11]. By contrast, we provide a software platform where users can develop their own probe databases. These databases (e-probes) are then used to determine pathogen presence/absence. MiFi^®^ crowd sources subject matter researchers to develop pathosystem-specific diagnostic tools that are then made available to diagnosticians to use on HTS data. This allows diagnostic clinicians to focus on wet lab techniques rather than having to learn how to undertake bioinformatic coding. Within MiProbe™ the Probe Tester software allows the e-probe developers to assess analytical sensitivity and hypothetical limit of detection of the e-probe set for a given microbe. Further curation and validation of the developed e-probe sequence set can be performed using a built-in script in MiProbe™. (2) MiDetect™ is the diagnostic side of the platform in which diagnosticians use e-probes to determine the presence or absence of pathogens/microbes in the metagenomic sequence data, using any computer with access to the internet [7,12,13,14,15,16,17].

The goal of this manuscript is to present the reader with suggested terminology and processes to validate HTS as a diagnostic tool. To exemplify how the validation process will work, this study presents the reader with a validation protocol using grapevine pathogens. The study includes comparisons of bioinformatic tools often used to analyze HTS data compared with MiFi^®^. Two basic performance characteristics defined for diagnostic assays are analytical sensitivity or limit of detection and analytical specificity. Here we will show how these two metrics are calculated for HTS data and can be affected by the bioinformatic tool used to analyze the data as well as the level of e-probe curation when using MiFi^®^.

## 2. Results

**Figure 1:**
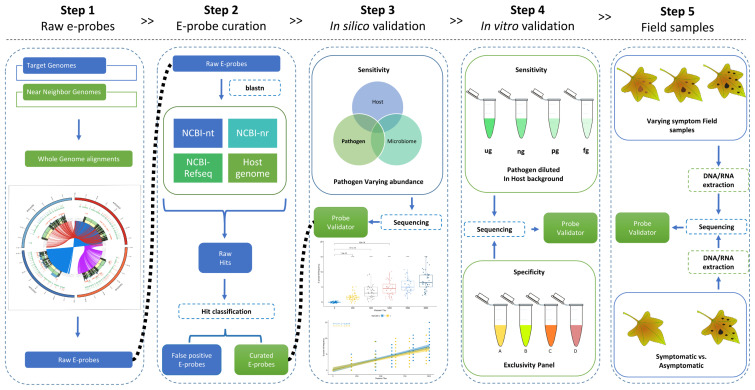
Full MiFi^®^ pipeline for the development, curation and validation of e-probes. Complete or partially assembled genomes are used in whole genome alignments of a target pathogen and near neighbor candidates. E-probes are curated in step two, using the National Center for Biotechnology Information (NCBI) reference databases. Subsequent steps (3–4) allow the user to do both in-silico and in-vitro validations to assess sensitivity and specificity. Finally, in step five, the e-probes are assessed using field samples with and without symptoms, and/or known positive/negative.

The model pathosystem used in this manuscript, grapevine pathogens, demonstrates how the MiProbe™ validation pipeline works. The steps for validation were followed as depicted in Figure 1. The scope includes validation in silico and the recommended pipeline for e-probe curation and establishment of performance characteristics metrics.

### 2.1. Data Selection and Raw E-Probe Design

The inclusivity panel for each (target) pathogen is comprised of concatenated sequences that include the complete or partial genomes of all/most isolates. The exclusivity panel or near neighbors, include the genomes of organisms often found in the matrix (grapevine) or taxonomically related to the target pathogen. The MiProbe™ software runs a massive parallel comparison of the panel files to generate a set of raw e-probes unique to the target. The same protocol was run on all grapevine pathogens using their respective target and near neighbor multi-fasta files. In the case of Grapevine Leafroll associated Virus 3 (GLRaV-3), a total of 15 raw e-probes of 60 nt in length were generated (Table 1).

### 2.2. E-Probe Curation

Some e-probe sequences may cause false positive hits if there is spurious alignment with a sequence in another organism. Curation is the process of exclusion, i.e., eliminating any e-probe sequences that have matches with other pathogens, inherent host microbiota or host genome (cross reactivity). E-probes generated on the twenty-three pathogens of grapevine originally had higher number of e-probe sequences (raw e-probes). The e-probe curation step undertaken with the NCBI nt/nr databases left a range of two to 14,236 unique e-probe sequences for pathogens of grapevine (Table 1). Curation of the e-probes decreased the total number of e-probe sequences by 25% to 50%. Specifically, for GLRaV-3, e-probe sequences were reduced from 15 to five after curation. Note, pathogen genome size impacts how many unique sequences will be identified. Viroids and viruses are much smaller than bacteria, which is reflected in the total number of e-probe sequences (Table 1). Closely related strains of pathogens might only have a few unique probes that differentiates between them.

### 2.3. In Silico Validation with Simulated High-Throughput Sequencing (HTS) Data

In silico validation determines analytical sensitivity also known as limit of detection (LoD) and specificity (i.e., false positives caused by reactivity and interference) of the e-probe sequences using simulated metagenomes [18]. For the purposes of bioinformatic analysis and diagnostics with MiFi^®^, we consider the analyte is a single pathogen-specific read. Therefore, all calculations are made in reference to the number of pathogen reads that a metagenome contains, and how many reads are detected by e-probes. The LoD can be represented as either absolute number (pathogen reads) or relative abundance. In this proof of concept, the LoD of GLRaV-3 is 400 pathogen reads, which transformed to relative abundance is 0.04% for 1 million reads metagenomes. A more exact LoD was calculated by serially diluting 400 pathogen reads, resulting in an LoD of 0.0102% or 102 pathogen reads (Table 1). Pathogen detection is achieved by quantifying total e-probe hits in simulated metagenomes. Each hit consists of two alignment metrics (percent identity and query coverage of the e-probe hit). The alignment metrics can be adjusted by the e-probe developer depending on the stringency needed.

**Figure 2:**
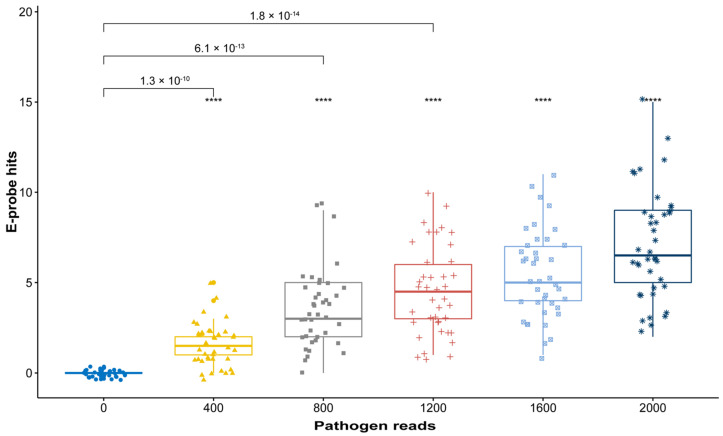
In silico pathogen (Grapevine Leafroll associated Virus 3, GLRaV-3) titer response with 15 e-probes. Boxplots depict e-probe hits distribution (*y*-axis) vs. a gradient pathogen titer (# reads of pathogen in the metagenome (*x*-axis)) in the simulated metagenome. With 15 e-probes, the *p*-value that determines true positives significantly distinguishes positive from the known negative control (*p*-value = 1.3 × 10^−10^). Statistical value meanings in the figure are ns: not significant, ****: *p* ≤ 0.0001. Boxplot colors represent a different pathogen read number in the metagenome and are labeled in the *x* axis.

A simulated metagenome is developed in silico by creating a mock metagenomic database with a host and a gradient of pathogen genome copies. In the following example, percent identities and query coverages were selected to be above 95% to classify a hit as positive. While comparing raw and curated e-probe hits, we can observe in Figure 2, how the raw e-probes can effectively discriminate positive control (400 pathogen reads) samples from a negative control (zero pathogen reads).

**Figure 3:**
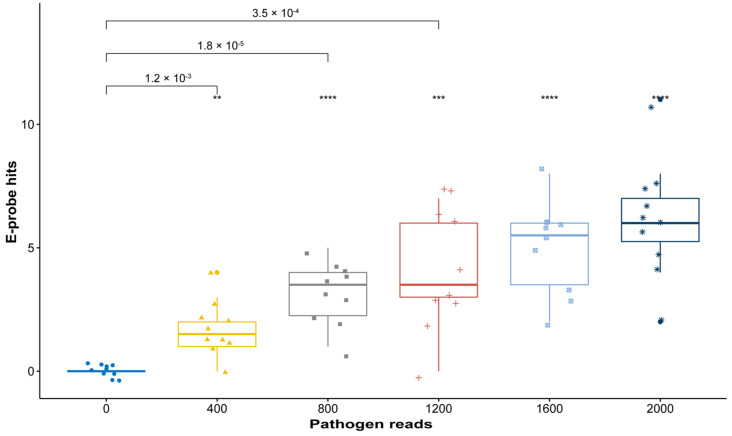
In silico response to increasing pathogen titer (GLRaV-3) with five e-probes. Boxplots depict e-probe hits distribution (*y*-axis) vs. a gradient pathogen titer (# reads of pathogen in the metagenome (*x*-axis)) in the simulated metagenome. It is observed that a *p*-value of 0.0012 is still able to distinguish a true positive from a known negative control. Statistical value meanings in the figure are ns: not significant, *p* > 0.05; **: *p* ≤ 0.01; ***: *p* ≤ 0.001 and ****: *p* ≤ 0.0001. Boxplot colors represent a different pathogen read number in the metagenome and are labeled in the *x* axis.

In simulated metagenomes having lower concentrations of pathogen genome we observed less false negatives after curation. In the GLRaV-3 model system, it is observed in Figure 3 that the average e-probe hits decreased for each of the serial dilution (fewer e-probes = fewer hits), yet it was possible to discriminate a positive sample from the negative control.

**Figure 4:**
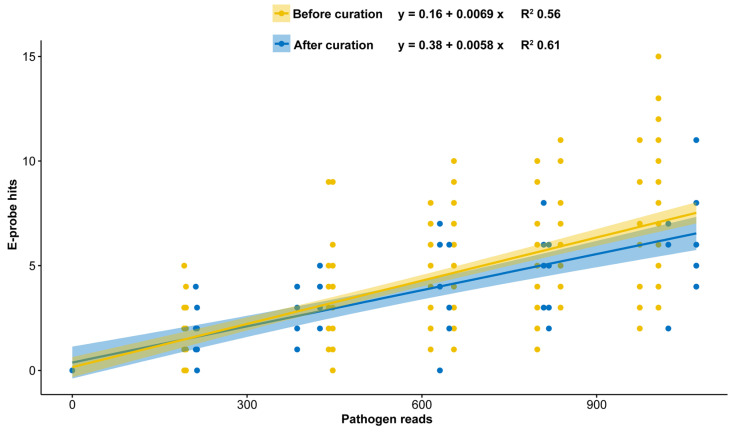
Grapevine Leafroll-associated Virus 3 (GLRaV-3) in silico e-probe semi-quantitative linearity comparison before and after curation. Metagenome simulated data (in silico) with GLRaV-3 at varying pathogen read abundances (with replicates) depicts how e-probes can be used for semi-quantitative analysis by having higher e-probe hits (*y*-axis) as the pathogen reads increase in the metagenome. The yellow dots and regression line show the quantitation model before e-probe curation (15 e-probes) and blue depicts the model after the e-probe curation (five e-probes). The intercept of the linear equation can be treated as a theoretical limit of detection (LoD).

In these simulations, the pathogen concentration is correlated with the e-probe hits. Therefore, when the pathogen titer reaches zero in the metagenome, any e-probe hits should be considered false positives (a sign of cross reactivity with the host) (Figure 4). Specificity may be increased when e-probes are removed based on spurious hits with other genomes or host. However, sensitivity is decreased when there are fewer e-probes available to query the metagenome. E-probe curation through the in silico curation process improves the quantitative capacity of the e-probe set, as observed in the R^2^ difference between raw and curated e-probes (Figure 4). Additionally, cross reactivity and interference tested with simulated multiple infections, yielded zero false positives, which shows the specificity of the curated e-probes for this pathogen panel (data not shown).

### 2.4. In Vitro Validation: Analytical Sensitivity and Specificity

In vitro validation is done by developing a serial dilution of the pathogen (positive control) in a background of host matrix/other matrixes. Each spiked sample is then put through nucleic acid extraction and library preparation for sequencing. The limit of detection must be determined by identifying the lowest pathogen concentration (copy number or nucleic acid concentration) where pathogen-specific e-probes can detect the pathogen consistently in MiDetect™. Most of these samples will likely vary in pathogen concentration, therefore, it is ideal that the same samples are analyzed using the gold standard (often polymerase chain reaction (PCR)) for comparison purposes. LoD is calculated as a probability of reliable detection when the analyte is rare in the metagenome [18].

### 2.5. Validation with Field Sample: Diagnostic Sensitivity and Specificity

In vivo validation (Figure 1) is undertaken using field samples that have been verified positive or negative for a specific pathogen via a gold standard method (often PCR). In this case, samples taken from various grape vines in Oklahoma did not show false positive results when using GLRaV-3 e-probes. These samples also were run using quantitative PCR (qPCR) and specific GLRaV-3 primers which showed a negative result. Therefore, the diagnostic specificity was 100% when comparing the GLRaV-3 with the gold standard.

### 2.6. Catalogue of Pathogen E-Probes for Other Hosts

The aforementioned validation pipeline has been replicated in other host–pathogen systems, which include economically important crops like citrus, blueberry, roses and wheat among others (Table 2). Similarly, the MiProbe™ platform has been used for the development of e-probes for animal and human pathogens which have been validated in silico using the MiProbe™ platform.

### 2.7. Comparison of Microbe Finder (MiFi) with Traditional Bioinformatic Tools Used for Diagnostics

Mapping to reference genome of a given virus using minimap2 took on average 35 s, which then multiplied by 23 pathogens of the grapevine is 805 s, or 13.41 min. On the other hand, the Basic Local Alignment Search Tool (BLAST) against the nt database took in average 5.3 h for each metagenome, in this case, only 20 metagenomes were analyzed due to the time and expense that it would take to analyze 100 metagenomes. Finally, using app.mifi.tech, we were able to get pathogen detection results within 10 min average for each metagenome for a set of 23 pathogens of grapevine.

When using data from the first Critical Assessment of Metagenome Interpretation (CAMI) metagenomes spiked with pathogen reads (500,000 total reads containing an average of 2493.46 pathogen reads), kraken2 was able to report most of the spiked grapevine pathogens within a period of six hours, however, Metaphlan3 failed to detect the grapevine pathogens. Building the Kraken2 standard database took five hours in a computer with 32 central processing units (CPUs) and 96 GB or RAM. The kraken2 data analysis was able to detect almost all pathogens of grapevine that were spiked in all metagenome complexity files within an average time of 2 h and 42 min. The pathogens that kraken2 failed to detect were *Candidatus Phytoplasma aurantifolia*, *Candidatus Phytoplasma solani*, Grapevine Leafroll-associated Virus 4(5), Grapevine Leafroll-associated Virus 4(9), Grapevine Leafroll-associated Virus 4(Pr) and Grapevine Leafroll-associated Virus 4(Car) (Supplementary Data 1).

Metaphlan3 database download took less than 10 min, however, the analysis took an average time of 1 h and 12 min. The analysis with Metaphlan3 was undertaken using 32 CPUs. The resulting output did not detect any of the spiked grapevine pathogens. However, high taxonomy ranks were reported, many at the family level, which are not useful for diagnostic purposes. Instead, it detected mostly bacteria consistent with previous results. The tutorial of metaphlan3 does mention that their database contains viruses, but it was unable to detect any of the viruses of grapevine.

Finally, the same spiked CAMI sets were uploaded and analyzed by MiFi^®^ and all the pathogens of grapevine selected for this analysis were detected. The analysis took an average time of 33 min when using the low- and high-complexity metagenomes. MiFi^®^ was able to detect all 23 pathogens of the grapevine (Supplementary Data 1).

## 3. Discussion

### 3.1. Data Selection and Raw E-Probe Design

Retrieving quality genomic data for e-probe design is a crucial step when creating the exclusivity and inclusivity panel. It requires the knowledge of a plant pathologist or a scientist familiar with the normal microbiome of the host and the phylogeny of other closely related taxa. Adding genomes that are too distantly related to the target genome in the exclusivity panel (near neighbors) often yields e-probe sequences with low specificity and potential false positive readings. On the other hand, selecting near neighbors too closely related to the target will generate very few e-probes, resulting in highly specific (strain level) discrimination, but reduced sensitivity as we have shown in Figure 2, Figure 3 and Figure 4. In the case of developing probes for differentiating closely related strains/species, the designer may have to make a few attempts with different variants of the near neighbor multi-fasta file to determine which near neighbor sequences generate the optimal number of e-probe sequences.

### 3.2. E-Probe Curation

E-probe curation to remove non-performing or non-specific sequences reduces diagnostic sensitivity and LoD. However, the remaining e-probes are often the most specific to detect only the pathogens in the inclusivity panel. The ability to adjust for analytical sensitivity and specificity on-the-go is a unique feature of the MiFi^®^ processes. PCR, enzyme-linked immunosorbent assay (ELISA) or other molecular-based diagnostic assays tend to be more static. It is imperative to find a good balance between sensitivity and specificity depending on the purpose of the assay. Greater sensitivity will be desirable if it is necessary to find pathogens in low titer, such as in cases where ‘freedom from’ infection must be certified. Specificity is most important when differentiation between an exotic vs. endemic pathovar is needed for regulatory purposes. For example, there are many strains of *Ralstonia solanacearum*, but only one strain is a potential invasive species and select agent [19]. Therefore, the MiFi assays can be adapted for the needs of the end user.

Total raw e-probe unique sequence numbers are usually correlated with the relative size of the pathogen. Viroids will have the fewest and true fungi will have many more initial probes. For a bacterial pathogen such as *Xylella*, unique e-probe sequences will number in the thousands (Table 1). Curating the sequences by removing a few e-probe sequences will not significantly alter sensitivity when the unique sequences are plentiful. With viroids and viruses, and with near neighbor strains, the balance becomes more nuanced.

### 3.3. In Silico Validation

Curated e-probes can be initially validated in silico to assess their analytical sensitivity and determine if improvements need to be made. The effect of reducing the number of e-probes during curation is often observed on the LoD. This is achieved by comparing e-probe hits of a negative control with a positive control having the lowest concentration. The *p*-values describing probability of differentiation between true negative and the lowest titer positive increased from 1.3 × 10^−10^ (Figure 2) to 1.2 × 10^−3^ (Figure 3) once e-probe numbers are reduced. The *p*-value increase is expected since the statistical power is reduced as the number of e-probes decrease. Part of the in-silico validation is to determine effective e-probe hit number to assess pathogen presence. Additionally, the hit number on e-probes has the potential to quantitatively infer the pathogen abundance [20]. Therefore, the in silico validation can serve two purposes, to determine e-probe LoD and quantitative efficacy. E-probe selection for quantification aims to improve the R^2^ of the linear response using a multiplicity analysis, whereby, e-probe sequences that are unresponsive to titer (the hit number does not increase in relation to pathogen abundance) can be identified and removed, improving quantitation (Figure 4). As we have shown, an e-probe sequence set curated to improve linearity by removing probe sequences may sacrifice analytical sensitivity and change the theoretical LoD by shifting the intercept (Figure 4). The y intercept is higher (0.16) before curation than after (0.38), meaning that differentiating between a true positive and a false negative becomes more demanding. The lack of cross reactivity and interference observed in the e-probes of grapevine pathogens denotes how this technology can help diagnosticians remove subjectivity generated by bioinformatic tools.

### 3.4. In Vitro Validation: Analytical Sensitivity (LoD) and Specificity

Analytical sensitivity and specificity are the performance characteristics of an assay relative to correctly detecting a pathogen in the host tissue matrix. When using HTS, the metagenome contains both the host and the pathogen. Sample collection can be a source of extreme variability, regardless of the diagnostic technique to be used. Additionally, the handling of sample material and preparation will have intrinsic biases depending on multiple factors. Sampling error is a known source of variability for almost all diagnostic assays. Sampling in plants that have heterogeneity in location of the pathogen in the tissue, or for which the pathogen goes into remission driven by environmental effects are notably problematic for any diagnostician. Also, even if a diagnostician is working with tissue that is clinically symptomatic, it is possible to have sampling effects during the wet lab sample preparation. HTS, like any molecular technique, requires a well validated sampling protocol and bench workflow, to assure that the pathogen is actually present in the final sample aliquot.

Each pathosystem and tissue matrix will require different methods for nucleic acid extraction and downstream molecular biology methods until reaching library preparation. All these are variables that must be taken into consideration while using this pipeline to validate e-probes. Additionally, we suggest that validation for in vitro analytical sensitivity must be accompanied by real-time PCR (or any other gold standard) results of the same samples being sequenced until the reliability of the test gains user confidence.

Finally, while in silico validation can provide theoretical diagnostic (analytical sensitivity and specificity) metrics of the e-probes, the metagenomic simulations are simplistic and do not represent the real composition of a plant microbiome, nor reflect the kinds of inhibitors that can interfere with sample preparation. The analytical specificity and sensitivity numbers might change after performing this analysis, since some false positive and negative e-probe hits are often observed, and e-probe sequences might require further curation.

### 3.5. Validation with Field Samples: Diagnostic Sensitivity and Specificity

The process of validating e-probes with field samples is critical to assess the breadth of diagnostic sensitivity and specificity of the assay. Field samples which have apparent symptoms and suggest the presence of the pathogen are sequenced and analyzed using curated e-probes. In parallel, the same samples should be analyzed using the gold standard method. Finally, diagnostic metrics are taken as tiers of in silico, in vitro and field sample validations. Ideally, the field samples should comprise different geographical areas across the country or world to confirm that the e-probe set is broadly specific.

Even though, there are guidelines for the validation of diagnostic tools in plant pathology [18], a guideline on tiers of validation for HTS-based diagnostic assays is still needed. In this manuscript we propose methods which can reach different levels of validation. It is up to the institution or researcher developing the HTS-based diagnostic assay to determine which level of validation can be achieved depending on availability of positive controls, funding, laboratory materials and field samples among others. The pipeline proposed here is not only valid for e-probes but could be modified to be used with other bioinformatic tools.

### 3.6. Comparison of MiFi with Traditional Bioinformatic Tools Used for Diagnostics

The use of bioinformatic tools for pathogen detection is limited to mapping to reference genomes and/or BLAST with the nt database. Even though mapping to reference genomes can be relatively fast, analyzing the output of Sequence Alignment Map (SAM) format requires the user to know bioinformatics. MiFi^®^ eliminates the subjectivity that SAM or BLAST output files create for users. Additionally, it reduces the requirement of a dedicated bioinformatician to analyze the HTS data. A few samples from the cross-reactivity analysis were used to compare MiFi^®^ with traditional bioinformatic tools for GLRaV-4. Results were ambiguous and it was difficult to discriminate between the different strains or isolates of GLRaV-4 when using BLAST or Kraken2 (Supplementary Data 1), while MiFi^®^ gave a clear answer within 30 min.

Although kraken2 generated promising results within a reasonable timeframe (~5 h), the results needed to be further parsed and analyzed to set thresholds to determine what is considered a positive sample [10]. That is an issue that any other bioinformatic tool has when being used for diagnostic purposes. Currently available tools are not able to produce an objective positive/negative result for pathogen detection. MiFi^®^ on the other hand generates unambiguous results within minutes to a couple of hours depending on the metagenome complexity. Similarly, Metaphlan3 relies on their own curated databases, and did not detect any of the grapevine pathogens [9]. A variety of other bioinformatic tools exist which allow read binning. The results generated by all available tools do not provide a clear-cut answer about pathogen presence, since they were developed for classification purposes, and interpretation is left to the operator. Additionally, the complexity of the metagenome is not directly related to its size (sequencing depth). Some metagenomes might be more complex composition wise, however be small in size. Using the CAMI data, we tested MiFi^®^ in both case scenarios. The sequencing depth is an important parameter that can improve the chances of finding the analyte (pathogen reads), therefore, knowing beforehand how deep one needs to sequence to effectively detect the pathogen is an issue that has been previously addressed by our team by developing a sequence calculator [21].

In conclusion, Metaphlan3 and Kraken2, BLAST and minimap2 are tools that can provide a broader perspective of the metagenome composition, as long as an experienced bioinformatician analyzes the data. However, the use of MiFi^®^ removes subjectivity when detecting pathogens and does not need specialized bioinformatics personnel. Additionally, MiFi^®^ speed relies in the simplicity of the e-probe databases, which are developed and validated for detection purposes and not exploratory.

### 3.7. Calculations of Sequence Depth for Assured Detection of an Analyte in a Complex Metagenome

A difference between HTS data generation for diagnostics and existing diagnostic tools is that in silico detection of a pathogen read based on an e-probe hit (and quality match index) in a metagenome is simply a probability function. Because we are working with sequence data, the computer search of that data is not subject to bias and will find the read if it is intact and present. It is just a matter of time and computing power. If the pathogen reads are abundant in the metagenome, such as when the sample is highly infected, the hits will happen within minutes. If the pathogen reads are few and far between in the metagenome, it will take more time, often up to 20 min, for the search algorithm to find them. The relative size of the non-pathogen genome (host + microbiome) and the relative size and abundance of the pathogen genome in the metagenome are the critical factors.

The sequence calculator is a tool that we developed to predict the total number of pathogen reads needed to effectively detect that specific pathogen within the sum of reads of the entire genome. The model uses as input known variables that can be obtained before a sequencing run is performed:

- Pathogen reads desired to detect
- Average read length (normal distribution)
- Probability
- Pathogen genome size (nts)
- Non-pathogen genome size, including host and co-habiting microbiome (nts)

When the equation is solved, it gives the number of total reads needed to detect a given number of pathogen reads [21]. This calculator helps the user know how deep a sequence needs to be for detection purposes, or whether some level of amplification may be needed.

## 4. Materials and Methods

MiFi^®^ MiProbe™ software contains tools for user/researchers to develop, curate and validate e-probes, which can be released as a diagnostic assay. The MiProbe™ full pipeline includes five steps to develop and validate e-probes for a pathogen of interest (Figure 1).

We used grapevine pathogens as a proof of concept to determine speed to detection, sensitivity, specificity and limit of detection. Whole genomes of 18 pathogens of grape were downloaded from public databases (Table 1). The complete list contained 13 viral species which comprised one DNA virus and 12 ssRNA(+) viruses. Additionally, five economically important bacterial pathogens of grapes were included. Data were retrieved from fully assembled or mostly complete genomes when multiple strains were available, alternatively, draft genomes were retrieved. All the genomes were retrieved using NCBI taxon IDs. In some cases, when the pathogen is considered important for diagnostics, strains were also included such as GLRaV-4 which is a viral species having strains in different viral genera: Ampeloviruses, Closteroviruses and Velarivurises.

### 4.1. Data Selection and Raw E-Probe Design (Step 1)

Target genomes refer to the collection of sequences that are grouped in the inclusivity panel of the diagnostic assay validation pipeline. The inclusivity panel should contain all/most isolates that belong to the taxonomy group for which we are developing e-probes. On the other hand, near neighbor refers to the exclusivity panel, which is a group or organisms that we do not want to detect using our diagnostic tool (e-probes). Therefore, for each pathogen, we retrieved all isolate genomes and concatenated them into a single multi-fasta file, named as target. For the near neighbor, we focused on other organisms often found in the grapevine phylosphere as well as taxonomically close relatives of the target. The near neighbors were also concatenated into a single multi-fasta file for each grapevine pathogen. For example, for the Grapevine Leafroll-associated Virus 3 (GLRaV-3), the near neighbor included the remaining pathogen genomes listed in Table 1 among others. Therefore, to generate e-probes for GLRaV-3, two files were uploaded to the MiProbe™ system, the target and the near neighbor multi-fasta files. After the target and near neighbors are in the system, the developer can select the length of the e-probe sequences to be generated, starting at 20 nt up to 120 nt. Based on previous findings, we suggest using short e-probe sequences (20–60 nt) for viruses and long e-probe sequences (60–100 nt) for bacteria, fungi and oomycetes [6,20].

### 4.2. E-Probe Curation (Step 2)

This stage is an alignment-based curation of the raw e-probe sequences with current publicly available databases (NCBI-nt, nr, refseq) where e-probe sequences that match anything but the target pathogen, a potential cause of false positives, are removed from the set [22]. In the curation step, e-probes causing false positive hits in nt/nr were removed. Here, each raw e-probe set is aligned to the NCBI databases. In cases where the number of e-probe sequences is small (such as for viroids or closely related strains), an exclusivity comparison with the host is recommended to eliminate any spurious matches. At the end of step 2, the developer has a set of e-probes that are very specific to the target.

### 4.3. In Silico Validation (Step 3)

Step 3 is an in silico validation with simulated samples (host genome) and different ratios of pathogen genome to assess limit of detection (LoD) and analytical specificity (cross reactivity and interference). The LoD is determined as the lowest pathogen concentration (reads) at which we can detect 95% of the positive samples. In this case, we had ten sample replicates, out of which, at least nine needed to be positive to determine the LoD. MiProbe™ will incorporate a built-in metagenome simulator to decrease metagenome uploads during validation, however, any third-party metagenome simulator can also be used. In our validation we used MetaSim and NanoSim [23]. For the example with grapevine, we simulated Illumina sequence data including the grape genome and GLRaV-3 at various concentrations. The simulations were capped at 1 million total reads. The simulated metagenomes were uploaded to MiFi^®^ Probe Tester via a metagenomes tab and queried with the new e-probe set. Using the raw data from the Probe Tester output, it is possible to generate a linear regression demonstrating the theoretical limit of detection. Semi-quantitative e-probes can be developed where curation (sequence elimination/inclusion) is based on determining which e-probe sequences are most responsive to pathogen gradient or titer. Response to pathogen titer is illustrated by the number of times a given e-probe hits on a matching sequence in the metagenome. At the end of step 3, the e-probe is validated in silico. E-probes may be placed into MiDetect™ for use by diagnosticians, with the understanding that the validation is preliminary.

To assess LoD and analytical specificity, NanoSim was used to generate a total of 10,000 reads from Oxford Nanopore Technologies. The simulations contained pathogens concentrations ranging from 4% to 0.005% (~400 pathogen reads) to 0.05% (~5 pathogen reads) and each concentration had 10 replicates. A total of seventy simulations performed allowed to assess LoD, cross reactivity and interference of the e-probes for each of the pathogens of grapevine in Table 1. For the cross-reactivity analysis, metagenomes that contained the other 22 grapevine pathogens (Table 1) were generated and the e-probes for the absent pathogen in the metagenome tested using MiFi^®^. The cross reactivity was performed at pathogen concentrations above the LoD, in our case our metagenomes had an average pathogen ratio of 0.1 in 0.9 of host (*Vitis vinifera* genome). Each cross-reactivity assay had 10 replicates, summing up to a total of 230 simulated metagenomes (Table 1).

### 4.4. In Vitro Validation (Step 4)

Step 4 is a more rigorous validation using in vitro samples spiked with the pathogen of interest. The spiking can be at the organismal, cellular or molecular nucleic acid level. The in vitro inoculated sample can be analyzed for analytical sensitivity and specificity using the e-probes in the Probe Tester software. Numbers regarding absolute pathogen concentration as copy number or nucleic acid concentration will be required.

### 4.5. Validation with Field Samples (Step 5)

Step 5 validation using known positive/negative field samples, verified against a gold standard assay, or where symptomatic and asymptomatic material is collected to create a gradient challenge to the e-probes; 100% clinical concurrence provides assurance that the e-probe set will perform as well as current methods.

### 4.6. Mi Detect

Validated e-probes are placed in the MiDetect™ host-specific library for use by diagnosticians following MiDetect™ guidelines. Metadata crediting the probe developer and institution and describing the level of validation are linked to each pathogen-specific e-probe set in a library. Third-party use of the e-probes may generate royalty for the developer if used in commercial settings. MiFi^®^ allows researchers and assay development scientists to create their own accounts to develop and validate e-probes. Additionally, if a publication has been generated from the development, it will be referenced in the e-probe metadata information.

Using MiFi^®^ allows researchers and diagnosticians to rapidly create databases to detect any type of pathogen or microbe. Using a small targeted database of e-probes to analyze genome sequence data decreases the time to detection from hours to minutes. The current platform sits on a mini-cluster and has a scheduler to increase the efficiency of jobs submitted by each user. Currently, MiFi^®^ can handle up to 800 jobs per week and up to 100 jobs in a single day of typical use, however we expect to expand scalability in the future. The functions offered in MiFi^®^ are coded in php, javascript and html, allowing the user to access the server remotely. Additionally, the system allows only compressed files to be uploaded, decreasing the time to upload HTS data. Once the data is uploaded, the user can select the genomes to develop e-probes in the case of MiProbe™. The MiFi^®^ MiProbe™ platform is being used by researchers who want to develop and validate e-probes for diagnostics and microbe detection.

### 4.7. Comparison of MiFi with Traditional Bioinformatic Tools Used for Diagnostics

Comparisons with traditional bioinformatic tools was performed using the AWS system, Ubuntu 18.04 O.S. and an EC2 instance with 4 CPUs and 8 Gb of RAM. The most utilized tools to detect pathogens in HTS were used, specifically minimap2 for mapping to the reference genome and BLAST to the nt database. A random sample of 70 metagenomes containing 100,000 minION reads simulated with NanoSim [23] was utilized. Each metagenome was analyzed as follows: 1. Mapping with minimap2 [24] to the reference genome; 2. BLAST with the nt database and 3. MiDetect was used at app.mifi.tech.

Data from the CAMI were retrieved for datasets of all complexities (high, medium and low) [25]. Each of the 10 CAMI metagenomes contained simulated reads of virus, bacteria and eukaryotic DNA. The pathogens of grapevine used in this manuscript were not included in the original CAMI metagenomes, therefore, we spiked the CAMI metagenomes with 500,000 simulated reads using NanoSim. Our simulated datasets had 10 replicates with similar pathogen abundances, which were incorporated into all CAMI metagenomic datasets (10) for a total of 100 metagenomes to be analyzed. MiFi^®^, Kraken2 v2.1.1 and Metaphlan3 v 3.0.6 were used to analyze the 100 metagenomes.

## 5. Conclusions

MiFi^®^ is a user-friendly and intuitive web application that is available online and updated to the most stable versions on a daily basis. The platform promotes rapid development and validation of e-probes for diagnostic purposes through the MiProbe™ process. MiFi^®^ has been successfully validated to work in plant and animal HTS data. Aditionally, MiFi^®^ eliminates the time-consuming bioinformatics learning curve, by making the developed tools available to the end users in an intuitive graphic user interface, allowing them to focus on the research and diagnostics instead of coding skills. MiFi^®^ requires subject matter experts that can be trained to develop highly efficient e-probes. Such training is provided on request by our research team at Oklahoma State University.

## Figures and Tables

**Table 1:** Relevant pathogens of grapevine used as a model to demonstrate MiFi^®^ as an interactive pathogen detection tool. E-probes are sets of unique 60 nt-long sequences. Minimum relative abundance of pathogen reads to detect (MRAD) is calculated by in silico simulations and in vitro experiments over a million total reads.

Species Name	NCBI Taxon ID	E-Probe Sequences	MRAD (LoD)
Grapevine Fanleaf Virus ^GFD^	12274	6	0.1272%
Grapevine Virus A ^RWC^	35288	5	0.0512%
Grapevine Virus B ^RWC^	35289	11	0.005%
Grapevine Leafroll-associated virus 1 ^GLD^	47985	16	0.0095%
Grapevine Leafroll-associated virus 2 ^GLD^	64003	10	0.0174%
Grapevine Leafroll-associated Virus 3 ^GLD^	55951	5	0.0102%
Grapevine Leafroll-associated Virus 4(4) ^GLD^	70177	9	0.0089%
Grapevine Leafroll-associated Virus 4(5) ^GLD^	71032	3	0.2521%
Grapevine Leafroll-associated Virus 4(6) ^GLD^	203168	2	0.3971%
Grapevine Leafroll-associated Virus 7 ^GLD^	217615	8	0.01%
Grapevine Leafroll-associated Virus 4(9) ^GLD^	184610	7	0.0105%
Grapevine Leafroll-associated Virus 4(Pr) ^GLD^	367121	9	0.01%
Grapevine Leafroll-associated Virus 4(Car) ^GLD^	659661	8	0.0094%
Grapevine Leafroll-associated Virus 13 ^GLD^	1815581	22	0.0017%
Arabis Mosaic Virus ^MD^	12271	7	0.1309%
Tomato Ringspot Virus ^YV^	12280	7	0.04%
Tobacco Ringspot Virus ^TRD^	12282	8	0.1304%
Grapevine red blotch-associated virus ^RBD^	1381007	2	0.4087%
*Xylella fastidiosa* ^PD^	644356	4034	0.0022%
*Agrobacterium vitis* ^CG^	373	14,236	0.0011%
*Candidatus Phytoplasma solani*	69896	83	0.0362%
*Candidatus Phytoplasma australiense*	59748	78	0.0257%
*Candidatus Phytoplasma aurantifolia*	180978	122	0.027%

GFD: Grapevine fanleaf degeneration; RWC: Rugose wood complex; GLD: Grapevine leafroll disease; MD: Mosaic disease; YV: Yellow Vein disease; TRD: Tobacco Ringspot decline; RBD: Red blotch disease; PD: Pierce’s disease; CG: Crown Gall; LoD: is represented as pathogen reads present in the metagenome.

**Table 2:** Hosts and number of pathogens with validated e-probe sets currently in the MiFi^®^ system.

Type	Host	Pathogens	Taxonomic Level
Plant	Grapevine	31	species, strain
	Citrus	43	Species, strain
	Rose	22	species
	Cucurbits	15	species
	Wheat	23	species
	Rhododendron	1	species
	Blueberry	3	strain
Animal	Swine gut microbiome	35	family
	Bovine respiratory disease complex	5	species
Human Pathogens on Plants	Food-borne pathogens	5	species

## Data Availability

The data presented in this study are available in Supplementary Data 1.

## Supplementary Materials

The following are available online at https://www.mdpi.com/2223-7747/10/2/250/s1, Supplementary data 1: Diagnostic results and computing speed comparisons of various bioinformatic pipelines and MiFi^®^ used to detect grapevine pathogens.

## References

[1] Paarlberg P.L., Hillberg A., Lee J.G., Mathews K.H. (2008). Economic Impacts of Foreign Animal Disease.

[2] Schaad N.W., Frederick R.D., Shaw J., Schneider W.L., Hickson R., Petrillo M.D., Luster D.G. (2003). Advances in molecular-based diagnostics in meeting crop biosecurity and phytosanitary issues. Annu. Rev. Phytopathol..

[3] Maree H.J., Fox A., Al Rwahnih M., Boonham N., Candresse T. (2018). Application of HTS for Routine Plant Virus Diagnostics: State of the Art and Challenges. Front. Plant Sci..

[4] Malapi-Wight M., Kumar L., Mollov D.S., Foster J. (2017). Implementation of next generation sequencing for high-throughput pathogen detection in sugarcane introductions grown in quarantine. Sugar J..

[5] Stobbe A.H., Daniels J., Espindola A.S., Verma R., Melcher U., Ochoa-Corona F., Garzon C., Fletcher J., Schneider W. (2013). E-probe Diagnostic Nucleic acid Analysis (EDNA): A theoretical approach for handling of next generation sequencing data for diagnostics. J. Microbiol. Methods.

[6] Espindola A.S., Schneider W., Cardwell K.F., Carrillo Y., Hoyt P.R., Marek S.M., Melouk H., Garzon C.D. (2018). Inferring the presence of aflatoxin-producing Aspergillus flavus strains using RNA sequencing and electronic probes as a transcriptomic screening tool. bioRxiv.

[7] Espindola A., Cardwell K.F. (2019). Microbe Finder (MiFi): Pathogen detection in metagenomic sequence data. Proceedings of the Plant Health.

[8] Caporaso J.G., Kuczynski J., Stombaugh J., Bittinger K., Bushman F.D., Costello E.K., Fierer N., Peña A.G., Goodrich J.K., Gordon J.I. (2010). QIIME allows analysis of high- throughput community sequencing data. Nature.

[9] Truong D.T., Franzosa E.A., Tickle T.L., Scholz M., Weingart G., Pasolli E., Tett A., Huttenhower C., Segata N. (2015). MetaPhlAn2 for enhanced metagenomic taxonomic profiling. Nat. Methods.

[10] Wood D.E., Salzberg S.L. (2014). Kraken: Ultrafast metagenomic sequence classification using exact alignments. Genome Biol..

[11] Huson D.H., Beier S., Flade I., Górska A., El-Hadidi M., Mitra S., Ruscheweyh H.-J., Tappu R. (2016). MEGAN Community Edition - Interactive Exploration and Analysis of Large-Scale Microbiome Sequencing Data. PLoS Comput. Biol..

[12] Zuniga L.P., Espindola A., Melouk H.A., Ali A., Cardwell K.F., Corona F.O. (2017). Detection of cucurbit viruses in Oklahoma combining EDNA with Multiplex RT-PCR coupled with High Resolution Melting. Proceedings of the 2017 APS Annual Meeting.

[13] Espindola A., Freire-Zapata V., Watanabe L.F.M., Corona F.O., Cardwell K.F. (2019). Detecting viruses and bacteria of grapevine with Microbe Finder (MiFi) and an Oxford Nanopore sequencer. Proceedings of the Plant Health.

[14] Espindola A. (2013). Massively Parallel Sequencing (Mps) As a Diagnostic and Forensic Analysis Tool for Important Fungi and Chromista Plant Pathogens. Ph.D. Thesis.

[15] Espindola A., Roy A., Mavrodieva V.A., Cardwell K.F. (2019). E-probe: A new diagnostic tool for detection of Dichorhaviruses associated with Citrus leprosis syndrome. Proceedings of the Plant Health.

[16] Bocsanczy A.M., Espindola A., Norman D.J., Cardwell K.F. (2018). E-probes development for rapid, sensitive and specific pathogen detection in blueberries. Proceedings of the ICPP Boston.

[17] Proano M.F., Espindola A., Garzon C.D. (2018). Detection of multiple oomycetes in metagenomic data by Using E-probe Detection of Nucleic Analysis (EDNA). Proceedings of the ICPP Boston.

[18] Cardwell K., Dennis G., Flannery A.R., Fletcher J., Luster D., Nakhla M., Rice A., Shiel P., Stack J., Walsh C. (2018). Principles of Diagnostic Assay Validation for Plant Pathogens: A Basic Review of Concepts. Plant Health Prog..

[19] Fegan M., Taghavi M., Sly L.I., Hayward A.C., Prior P., Allen C., Elphinstone J. (1998). Phylogeny, Diversity and Molecular Diagnostics of Ralstonia solanacearum. Bacterial Wilt Disease: Molecular and Ecological Aspects.

[20] Espindola A., Schneider W., Hoyt P.R., Marek S.M., Garzon C. (2015). A new approach for detecting fungal and oomycete plant pathogens in next generation sequencing metagenome data utilising electronic probes. Int. J. Data Min. Bioinform..

[21] Espindola A., Habiger J., Cardwell K. (2019). Metagenome sequence calculator for effective pathogen detection. Proceedings of the Plant Health.

[22] Camacho C., Coulouris G., Avagyan V., Ma N., Papadopoulos J., Bealer K., Madden T.L. (2009). BLAST+: Architecture and applications. BMC Bioinform..

[23] Yang C., Chu J., Warren R.L., Birol I. (2017). NanoSim: Nanopore sequence read simulator based on statistical characterization. Gigascience.

[24] Li H. (2018). Minimap2: Pairwise alignment for nucleotide sequences. Bioinformatics.

[25] Sczyrba A., Hofmann P., Belmann P., Koslicki D., Janssen S., Dröge J., Gregor I., Majda S., Fiedler J., Dahms E. (2017). Critical Assessment of Metagenome Interpretation-a benchmark of metagenomics software. Nat. Methods.

